# Editorial Notes

**Published:** 1920-09

**Authors:** 


### Editorial Notes.

**The Report of the Consultative Council under the Ministry of Health.**

In issuing their Interim Report the Consultative Council have wisely given the country generally as well as the Medical Profession an opportunity for timely consideration of the general conclusions of the Council set up to advise the Ministry of Health in the difficult and complex problem of improving the Public Medical Services.

There is no longer any doubt that the Medical Insurance Act has failed to meet the urgent necessities of the day even in its limited scope, and the failure which was feared and widely predicted by the medical profession has become only too obvious. We are convinced that if the medical profession had been called in to advise the legislature through its responsible organisations, and the advice followed, recent legislation would have proved of infinitely greater value and far less costly ; and we therefore feel that in setting up an Advisory Council the newly-constituted Ministry of Health has displayed prescience and an earnest desire to seek the assistance and co-operation of medical practitioners in shaping their policy for the public welfare.

It is of the utmost importance that every medical practitioner should actively interest himself in the proposals to organise and develop the national medical services, and also endeavour to interest and to guide those who are responsible for the organisation of hospitals and all other existing institutions for the prevention and treatment of disease.

To this end the President of the Bath and Bristol Branch of the British Medical Association invited Lord Dawson to Bristol to meet representatives of all the more important hospitals and other cognate organisations. Our Local Notes afford a short summary of the speeches of Lord Dawson and others, firstly at the luncheon and secondly at a special meeting of the branch of the British Medical Association (see p. 187).

But it is not enough to become acquainted with the proposals that are afoot. We as a medical profession should take an active share in promoting, and perhaps modifying, these schemes, in guiding the legislature and the philanthropists, and in directing the actions of the representative medical organisations, so that the best and most fruitful results may eventually emerge. Individual criticisms are of little avail, and are apt at best to be destructive. What we need is constructive organisation, and this is alone possible by utilising and strengthening existing medical organisation so as to make it representative of the medical profession as a whole. The needs to be met are national needs, the co-ordination of the medical services must be a national and combined movement. But a lead in the right direction is afforded by the Gloucestershire scheme which Dr. Middleton Martin has initiated. If this be taken as a nucleus and developed, it may become sufficiently large and complete to demonstrate how under the guidance of medical men the State may secure a medical service which infuses the essential spirit of the existing voluntary system into a larger and more adequate national service, and which does not destroy or replace existing institutions, but develops and evolves the work that they have hitherto carried out.

But it will have to be recognised that " *one cannot make*

*bricks without straw,*" and that considerable grants must be made towards the cost of this or any other scheme for the public benefit. The medical profession as a body is woefully paid for its services, in a measure due to the practitioner's readiness to shoulder medical burdens for the public welfare. The medical practitioners deserve a larger recompense for their work ; but every step forward in the development of the provisions owed its inception to their advocacy, hence there has grown up a feeling that what they have called into being they must work and leave it to a grateful public to accord a fair remuneration. The birth of the great hospital movement, the development of the Medical Officer of Health, the Venereal Clinics, and finally the inception of the Ministry of Health, all are the outcome of medical advocacy.

It is the duty of representative medical organisation, *e.g.* the British Medical Association, to press for adequate remuneration for the general practitioner and other medical men whose work is the basis of the Middleton Martin or any other scheme. Voluntary medical service so generously accorded to the voluntary hospitals is splendid, so long as the medical practitioner can afford to give it and the patients are fit and proper persons to receive unpaid service ; a paid hospital service, even on a meagre basis, is at least a commercial transaction. But to fall between two stools, to accept a pittance as the price of medical services in voluntary hospitals for the relatively well-to-do would be folly, and only lower the economic position of every practitioner. Let us equally guard against the general practitioner being lured into an analogous position by a scheme which, however excellent in its scope and organisation, fails to provide adequately the labourer's hire.

**A Paying Hospital for Bristol.**

In former issues we have already directed attention to the need for a "paying hospital" to provide for the large and growing class of patients whose means render them u nsuited for treatment in charitable institutions, and yet who are not in a position to pay the normal fees for surgical operations or other special medical attendance in addition to the necessarily high charges now obtaining in first-class nursing homes.

The voluntary hospitals were founded for patients who are too poor to pay for medical attendance adequate to the necessities of their complaint, and the professional attendance is given freely and without any fee. It is not well that those who can afford to pay acceptable fees for medical or surgical attendance and the cost of their maintenance should be compelled to encroach on what is provided for their poorer neighbours, particularly at a time when the long waiting lists at these hospitals prove how inadequate is the present hospital accommodation. It follows that there is a hiatus in existing institutions for the class between the well-to-do and the poor, for whom other than existing institutions are required. But further, it is not right that a patient who is well-to-do should not have access to the necessary scientific and clinical equipment, similar to that now obtaining in a modern hospital for the poorer classes, solely because he is able to pay for them.

Members of the Medical Faculties of the Royal Infirmary, General Hospital, Royal Hospital for Children, and of the Eye Hospital met on September 22nd and passed the following resolution: "A completely equipped hospital is urgently needed in Bristol for all classes of patients who are able to pay for medical and surgical attendance in addition to charges for maintenance." It was felt that such a hospital should be available for the patients of all physicians and surgeons, both general and special, and that it was not desirable for the voluntary hospitals to provide for patients who could thus provide for themselves.

